# RNA Sequencing in Disease Diagnosis

**DOI:** 10.1146/annurev-genom-021623-121812

**Published:** 2024-08-06

**Authors:** Craig Smail, Stephen B. Montgomery

**Affiliations:** 1 Genomic Medicine Center, Children’s Mercy Research Institute, Children’s Mercy Kansas City, Kansas City, Missouri, USA; 2 Department of Biomedical Data Science, Department of Genetics, and Department of Pathology, Stanford University School of Medicine, Stanford, California, USA

**Keywords:** transcriptomics, RNA sequencing, genetic disease

## Abstract

RNA sequencing (RNA-seq) enables the accurate measurement of multiple transcriptomic phenotypes for modeling the impacts of disease variants. Advances in technologies, experimental protocols, and analysis strategies are rapidly expanding the application of RNA-seq to identify disease biomarkers, tissue- and cell-type-specific impacts, and the spatial localization of disease-associated mechanisms. Ongoing international efforts to construct biobank-scale transcriptomic repositories with matched genomic data across diverse population groups are further increasing the utility of RNA-seq approaches by providing large-scale normative reference resources. The availability of these resources, combined with improved computational analysis pipelines, has enabled the detection of aberrant transcriptomic phenotypes underlying rare diseases. Further expansion of these resources, across both somatic and developmental tissues, is expected to soon provide unprecedented insights to resolve disease origin, mechanism of action, and causal gene contributions, suggesting the continued high utility of RNA-seq in disease diagnosis.

## INTRODUCTION

RNA sequencing (RNA-seq) has continued to evolve as a molecular assay since its introduction in 2009 (116). RNA-seq characterizes and quantifies sequences of genetic code resulting from the process of transcription in a particular cellular context (77), providing unique insights over DNA sequencing in understanding the relative expression levels of gene products (32), transcript isoform usage (69), and alternative splicing (71) underlying disease phenotypes. Sequencing of mRNA—the code for amino acids, the building blocks of proteins—is the most common application of RNA-seq, achieved by applying a polyA enrichment process to total RNA isolated from donor blood or tissue samples (125). However, abundant protocols are available for other RNA molecules of interest, such as RNAs involved in gene regulation (101).

**Figure 1:**
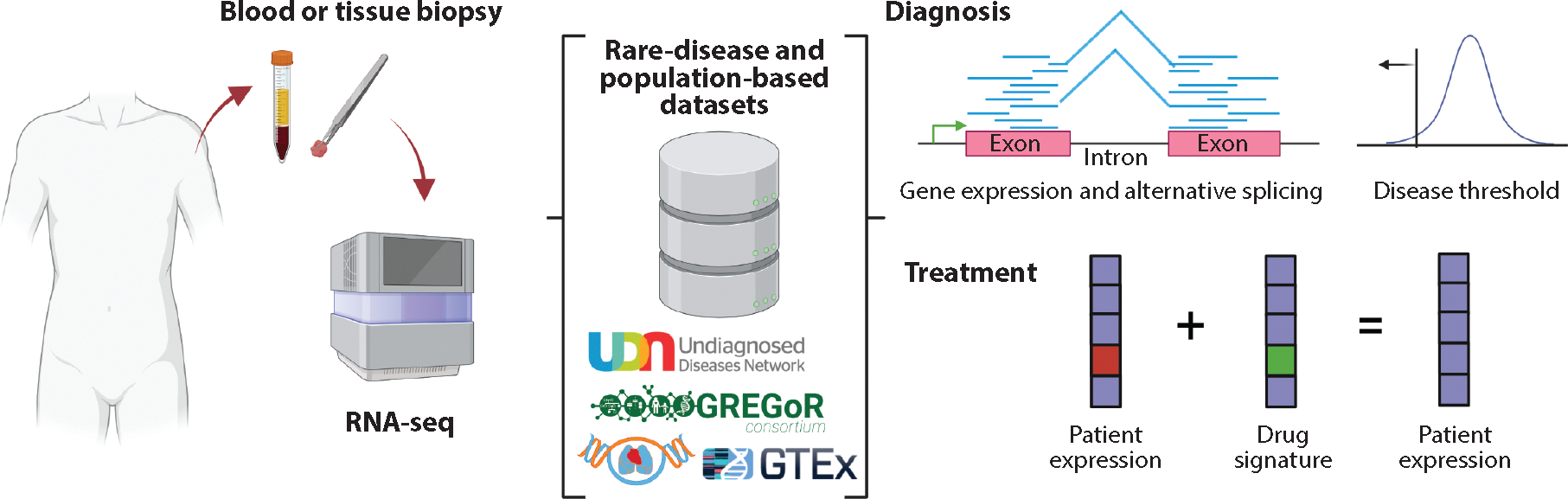
RNA-seq in disease diagnosis and treatment. A patient’s biospecimen is subject to RNA-seq and subsequently deposited in or compared with rare-disease and population-based RNA-seq datasets. Outlier gene expression or alternative splicing can inform an underlying pathogenic effect to assist with diagnosis, and comparison with drug signatures that may restore normal molecular function provides an opportunity to identify treatment strategies. Abbreviations: GREGoR, Genomics Research to Elucidate the Genetics of Rare Diseases; GTEx, Genotype–Tissue Expression; RNA-seq, RNA sequencing. Figure adapted from images created with BioRender.com.

In this review, we present an overview of established and emerging technologies, protocols, and analytical strategies for the generation and processing of RNA-seq data, with a focus on rare-disease applications (see Figure 1). We highlight state-of-the-art approaches using short-read sequencing of bulk RNA samples, as well as newer technologies that are rapidly increasing our ability to resolve the complexities of transcriptomic landscapes through the use of long-read, single-cell, and spatial transcriptomics innovations. We also discuss applications of RNA-seq to the diagnosis of rare diseases, as well as in identifying RNA biomarkers of disease progression and potential therapeutic targets. We further outline how the increasing availability of population-scale multiomic resources is providing better understanding of gene dosage effects and mechanisms of action of candidate disease variants. Overall, we outline the many impacts of RNA-seq since its introduction and show how this technology will continue to offer unprecedented insights into transcriptomic profiles of health and disease.

## ADVANCES IN RNA-SEQUENCING APPROACHES

Approaches for RNA-seq using next-generation sequencing technologies are now well established. Longer read lengths and increased depth of sequencing have steadily provided deeper resolution of transcript-level expression using Illumina platforms (7, 20, 23, 98). At present, paired-end RNA-seq using an Illumina MiSeq can generate 2×201-bp data, enabling contiguous sequencing across multiple internal exons of a gene. However, this approach is more cost-prohibitive, and most RNA-seq applications focus on 2×150-bp data provided by higher-throughput machines. Third-generation platforms from companies such as PacBio and Oxford Nanopore now also provide the opportunity to significantly extend sequencing of cDNA molecules with average read lengths ranging from 700 to 5,000 bp and beyond (38, 39, 58). Such platforms still present several biases, including in their coverage, sequence, and higher base error rates (2, 79). However, continued advances are increasingly allowing for direct RNA-seq that, unlike conventional RNA-seq, also supports the measurement of RNA modifications and potential analysis without requiring a genome reference (27, 50, 114).

Methods for processing RNA-seq libraries have also evolved to enable more routine depletion or enrichment of specific gene products. Routine use of polyA enrichment and ribosomal RNA depletion enables capturing full-length mRNAs and removing highly abundant species that can dominate RNA-seq libraries (83, 125). For specific tissues, additional highly abundant RNAs may be selectively depleted. For example, in blood RNA-seq, globin depletion further removes the large quantities of globin RNAs that can dominate a sequencing library (76). The majority of these approaches use either magnetic bead pull-down or degradation with RNase H (1). The Depletion of Abundant Sequences by Hybridization (DASH) protocol uses the Cas9 nuclease to cut specific sequences and limit their amplification to remove unwanted sequences (44). The benefit of these specialized RNA-seq approaches will be to provide new opportunities to focus on low-abundance and potentially more tissue-restricted transcripts involved in disease. For example, the *DMD* gene involved in muscular dystrophy has been surveyed for splice defects in patient muscle (24); in Genotype–Tissue Expression (GTEx) Project data (43), *DMD* is expressed at a TPM (transcripts per million) of ~20 in muscle samples but is also expressed at a low level in blood, at a TPM of ~0.03. In addition, targeted RNA-seq shows comparable opportunities in rare-disease settings to evaluate expression of a disease-relevant gene panel (8). This suggests that canonically tissue-specific expression may be more observable with depletion or enrichment approaches for RNA-seq, thereby avoiding the need for invasive patient biopsies.

Complementing advances in RNA-seq, the rapid advances in single-cell and spatial RNA-seq have opened new opportunities for identifying molecular features in health and disease states (70, 90, 107, 118). Primarily, the advantages of single-cell approaches are their ability to quantify RNA in cells that are hard to detect due to low abundance or transience in bulk samples or, further, are misrepresented by averaging RNA quantification across cells. A representative example of the power of this approach has been the characterization of nearly 300,000 immune cells from 367 prenatal samples to detect microglia-like cells and their subtypes outside the central nervous system and their respective developmental dynamics (117). Comparable analyses using bulk RNA-seq would rely heavily on cell sorting and computational deconvolution, significantly increasing time and expense. Spatial transcriptomics takes this approach one step further to quantify cells in situ and their gene regulatory programs in the context of their local environment and interactions. For example, application of spatial transcriptomics in Alzheimer’s disease has helped to identify local gene expression programs proximal to amyloid plaques (18). Spatial transcriptomics has been further applied to map degenerative processes in spinal cords of amyotrophic lateral sclerosis (ALS) mouse models and humans (73). The application of these approaches is only expected to grow, given their potential to identify cellular transitions to disease states and more complex, regional patterns of cellular heterogeneity.

## USING RNA SEQUENCING FOR BIOMARKER DETECTION IN RARE DISEASES

Transcriptome profiling provides an opportunity to identify RNA biomarkers that inform disease progression, disease subtypes, and response to therapy (9). RNA biomarkers can further aid in repurposing novel drugs or therapies for a wide range of genetic diseases—several studies have screened for drugs that show evidence of reverting a disease expression profile to a healthy profile (30, 56, 119). In comparison with biomarkers for diseases like cancer, RNA biomarkers for rare diseases are scarce as genetic information is considered sufficiently diagnostic; however, in therapeutic development, genetic data alone are unable to monitor patient progression through any intervention.

We highlight specific rare diseases that provide representative examples of RNA biomarker discovery attempts. In Huntington’s disease (HD), early attempts at finding RNA biomarkers in humans leveraged shared changes in skeletal muscle of an HD animal model and human patients to identify a shared muscle gene expression signature (104). Notably, this signature represented multiple factors, including endocrine-system changes, muscle-specific polyglutamine effects, and aberrant signaling from the central nervous system, highlighting the ability to identify path-way insights into preclinical therapeutic responses. However, obtaining RNA biomarkers from muscle has a higher patient burden than blood-based RNA assays, and several studies in HD have compared blood to brain RNA signatures to assess their commensurability for monitoring progression (11, 75, 94). Despite a larger number of candidate RNA biomarkers discovered in blood over the past decade, including *mHTT*, few have robustly replicated across studies, and none have been used as routine clinical tests for HD (74), suggesting that even in well-studied rare diseases with a known genetic etiology, identification of robust RNA biomarkers remains challenging.

RNA biomarker discovery in cystic fibrosis (CF) provides a separate representative example of the potential of RNA biomarkers to identify signatures of treatment response. One such effort by Jiang et al. (52) reported on the discovery of a six-gene expression signature in neutrophils from RNA-seq of 16 patients that separated patients between admission and after treatment. However, as with many biomarker discovery studies, the study had a reduced sample size, and its impact was mitigated by patient-level variance in disease-relevant responses. To reduce variance, Sun et al. (106) focused on a larger, genetically homogeneous population of CF patients with at least a G551D mutation in the *ABP2* gene during treatment with ivacaftor, a CF potentiator approved by the US Food and Drug Administration (FDA) for carriers of G551D. Notably, they identified a pretreatment signature that separated “good” from “moderate” response, highlighting the potential for an RNA biomarker to classify therapeutic response.

As of November 2022, several dozen studies are listed at ClinicalTrials.gov for “RNA biomarker” or “gene expression biomarker.” However, despite advances in RNA-seq and single-cell RNA-seq, there remain multiple impediments to translating an RNA biomarker to the clinic (13). Two major roadblocks to more routine use of RNA-seq in clinical settings have the potential to be mitigated soon. First, multiple countries have now approved population-based RNA-seq through their health systems and biobanks. Future increased availability of population-scale data linking transcriptome data to clinical measurements will provide large-scale normative reference data and standards for RNA-seq that can accelerate robust discovery of RNA biomarkers. Second, RNA-based therapeutics for rare diseases such as antisense oligonucleotides (ASOs) in spinal muscular atrophy have recently provided new opportunities to treat rare diseases (91). These therapeutic approaches require an increased need to monitor the transcriptome for successful intervention, the cell-type specificity of their impact, and durability over time.

RNA biomarkers are also providing new opportunities for identifying or repurposing novel drugs. The National Institutes of Health Library of Integrated Network-Based Cellular Signatures (LINCS) consortium has provided drug-induced transcriptome signatures across a range of cell types (105). Such signatures, when intersected with robust RNA biomarkers, can nominate candidate therapeutics that revert from a disease to a normal expression profile (30, 56, 119). However, the major challenge with this approach has been that a disease expression profile may also include protective, homeostatic factors (88). A new method that investigates reversion from genetically predicted expression has the potential to minimize these issues and has demonstrated potential for broad identification of repurposing candidates (119). As these data resources and methods continue to develop, RNA-based biomarkers, whether directly observed or genetically predicted, can be increasingly screened for candidates for drug repurposing in individuals with a broad range of genetic diseases.

## RNA SEQUENCING IN RARE-DISEASE DIAGNOSIS

RNA-seq has been increasingly applied to uncovering dysregulated molecular profiles underlying rare-disease patient cases. In one of the first large-scale applications of this approach, Cummings et al. (24) reported a 35% diagnosis rate using RNA-seq of affected tissue biopsies in a cohort of 50 patients with rare muscle disorders. Their approach further aided in interpreting a de novo intronic mutation in *COL6A1* that explained 25% of cases in an external collagen VI–related dystrophy patient cohort. This study also demonstrated the utility of using external matched tissue control cohorts, in this case skeletal muscle samples from the GTEx consortium (42).

Previous studies have also demonstrated how RNA-seq can aid in prioritizing candidate rare-disease variants from DNA sequencing. In a pediatric muscular dystrophy case, Gonorazky et al. (40) used RNA-seq to show how a deep intronic variant in *DMD* leads to a novel exon. Applying this approach more broadly, RNA-seq of blood, fibroblast, muscle, or bone marrow across a cohort of 113 rare-disease cases of diverse pathophysiologies increased diagnosis from 31% to 38% compared with DNA sequencing alone (60). A subset of cases diagnosed only through the addition of RNA-seq included neurology cases, diagnosed using patient blood samples. This showed that—at least for a subset of cases—blood can be used as a proxy for disease-affected tissues, which is important where affected tissue is not clinically accessible or biopsy would carry high risk. Frésard et al. (36) systematically assessed the diagnostic potential of RNA-seq of blood samples from 94 patients across 16 disease categories. Importantly, they found that 70.6% of OMIM rare-disease genes were expressed in blood, and that rate was similar in OMIM disease subcategories such as neurological, musculoskeletal, hematological, and ophthalmological diseases. Across the full cohort, a diagnosis was returned for 7.5% of cases, with a further 16.7% where RNA-seq improved candidate causal genes through the addition of transcriptomic dysregulation evidence.

Although blood has typically been the most used tissue to date in RNA-seq applications to rare-disease diagnosis, the expression of known rare-disease genes in this tissue captures only 60–80% of genes across OMIM disease categories (36). However, expanding to other clinically accessible tissues (defined as whole blood, Epstein-Barr virus–transformed lymphocytes, skeletal muscle, and skin-derived fibroblasts), studies using GTEx data show that more than 90% of OMIM disease genes are expressed in at least one clinically accessible tissue (121), increasing the potential for finding pathogenic variants with observable impacts on transcriptomic signatures. Demonstrating this approach, Kremer et al. (57) used RNA-seq of skin-derived fibroblasts in a mitochondriopathy patient cohort, reporting a 10% diagnosis rate from assessing aberrant gene expression, splicing events, and allele-specific expression. Furthermore, human induced pluripotent stem cells (hiPSCs)—most commonly generated from skin-derived fibroblasts—also express known rare-disease genes (as listed in OMIM) not expressed in blood (3, 10, 15). Bonder et al. (10) used a population-scale cohort of hiPSCs to model the transcriptomic impacts of candidate rare disease in variants in a hereditary cerebellar ataxia cohort proximal to *CACNA1A*, a gene broadly expressed primarily in the cerebellum and with absent or extremely minimal expression in nonbrain tissues. Furthermore, hiPSC programming protocols are available that promote lineage-specific differentiation, with application to the recapitulation of developmental trajectories in brain (4) and heart (21) tissues, among others.

Recent technological advances have enabled long-read RNA-seq approaches applied to characterizing full-length gene isoforms, improving the resolution possible with short-read technologies. Full-length transcript isoform sequencing resolves full mRNA molecules and as such provides higher resolution than short reads, which require a subsequent computational mapping step that can be error prone. Long-read approaches typically use PacBio or Oxford Nanopore platforms to measure full-length transcripts. For example, Stergachis et al. (103) utilized PacBio Iso-Seq to identify alternative splicing impacts in Charcot–Marie–Tooth disease. In a study by Farrow et al. (34) using the same technology, the discovery of a novel splice site solved a patient case of caseinolytic peptidase B (CLPB) deficiency where clinical testing using genome sequencing was negative. Similarly, using nanopore full-length RNA-seq, Helman et al. (45) identified a cryptic exon underlying a rare mitochondrial disorder.

Long-read approaches have further enabled improved reference genome assemblies for the analysis of previously unmappable RNA-seq reads. For example, one such assembly—Telomere-to-Telomere (T2T) Consortium CHM13—reports almost 2,000 novel genes, including 99 predicted as protein coding, compared with GRCh38 (86). Long-read assemblies, combined with RNA-seq, is a particularly useful strategy in resolving and modeling the impact of repeat expansions within genes (79) and longer-range segments overlapping multiple gene regions, such as the rare-disease gene *REXO1L* contained within the 1-Mb variable number tandem repeat (VNTR) region (46).

## COMPUTATIONAL APPROACHES FOR USE OF RNA SEQUENCING IN RARE-DISEASE CONTEXTS

Ongoing development of computational approaches has increasingly enabled use of RNA-seq approaches. Beyond gene expression levels, accurate quantification of splicing (66, 111, 113), alternative gene structure (64, 123, 126), RNA modifications (65), and allele-specific expression (6, 17, 59) all provide insight into normal and disease-relevant molecular activities. In the context of rare-disease diagnosis, the ability to measure normal and aberrant splicing has enabled the increased ability to classify pathogenic noncoding alleles (31) and identify potential therapeutic targets (16). These approaches are also rapidly being advanced with the availability of computational tools such as SpliceAI and Pangolin (49, 124) and via the reprocessing of hundreds of thousands of RNA-seq files to identify prior evidence of splice events (25). Computational approaches that measure allele-specific expression from RNA-seq data have further provided insight into the mechanisms of nonsense-mediated decay and provide candidate therapeutic targets (22, 84, 92, 109). Further single-cell pipelines that enable improved detection of individual cell types have enabled profiling of rare cell types or differentiation trajectories that underlie rare-disease effects (47, 48, 82, 108). Single-cell approaches offer further unique opportunities to compare wild-type with disease cells in the same patient as a result of mosaic expression of the candidate disease allele (19).

Assuming a FASTQ file output from an RNA-seq run, several computational processing steps are necessary to prepare data for downstream analysis. Mapping aligns mRNA reads to a reference genome; multiple software packages are available, including Spliced Transcripts Alignment to a Reference (STAR) (28) and Minimap2 (63). Mapped reads can then be quantified at the gene level, or further at the level of transcript isoforms and individual exons (26, 62, 67, 89, 110). Additional molecular phenotypes obtainable from RNA-seq include known or novel splicing variation quantification (66), alternative polyadenylation, and—if genetic data are available—allele-specific expression (112).

Various sources of technical noise, combined with intrinsic transcriptomic heterogeneity across genes, can adversely affect downstream analyses that use RNA-seq data. To alleviate this issue, a common approach is to implement a noise correction algorithm on filtered, transformed, and normalized data. Filtering is performed to remove genes expressed at low levels across the study cohort and typically uses empirical thresholds (96), which controls for sparseness in individual observations and removes genes where low expression might bias downstream analyses. Log transformation and standardization for variance stabilization accounts for differences in the magnitude of expression across genes (127). Approaches implementing technical noise correction discover latent variables capturing shared patterns of expression variation across individual samples, contributed by experimental factors such as library preparation and sequencing batch (61, 102).

Comparing transcriptomic readouts within or between individual samples enables the identification of outlier molecular signatures. These outlier signatures potentially result from proximal large-effect variants impacting normal gene function. Outlier signatures at the level of total gene expression are typically ascertained through *Z*-score transformation (36, 41), enabling a fixed outlier threshold to be set across genes. As a further step, to separate genetic from technical/nongenetic outliers, one potential strategy is to summarize individual outlier counts across the full dataset—for example, by calculating the proportion of outlier genes per individual across all outlier calls (35). Individuals contributing a relatively higher proportion of outlier calls might be reflective of uncorrected technical noise and can be removed from further analysis. An alternative approach is Outlier in RNA-Seq Finder (OUTRIDER) (12), which identifies gene expression outliers after first accounting for confounding variation using expression correlation learned by an autoencoder. Relevant to additional molecular phenotypes obtainable from RNA-seq, Analysis of Expression Variation–Dosage Outlier Test (ANEVA-DOT) (81) can characterize allele-specific expression to measure the magnitude of deviation in equilibrium of gene expression between paternal and maternal haplotypes for a candidate pathogenic variant informed by a reference/control population. For identifying splicing events, LeafCutter (66) uses mapped split reads to identify intron removal and indicate splicing junctions. The Detection of RNA Outliers Pipeline (DROP) (122) provides a unified workflow to detect aberrant expression, splicing, and monoallelic expression. Increasingly, the predicted molecular effects can be tested when suspect expression or splicing-modulating variation is identified through patient assays that compare isogenic cell lines through gene-editing-based correction of candidate disease variants (99).

There are multiple opportunities for future development of computational methods. Downstream of accurate quantification of molecular phenotypes from RNA-seq data, analysis strategies for comparison with reference/control resources are required to identify and interpret candidate disease variants linked to outlier molecular profiles. Trio RNA-seq of proband and parents has begun to aid in modeling transcriptomic impacts of candidate disease variants (60). However, tools that routinely integrate family-based transcriptomics in disease diagnosis are largely missing. Further, approaches like Watershed that integrate genome annotation and personal transcriptome profiles are providing new opportunities to prioritize specific rare variants (35). But unlike the multiple tools that predict variant effects from sequence alone, there are limited comparable approaches that integrate personal omics to identify signatures of pathogenic variants. As an example, dynamic, personal transcriptome signatures will need to be analyzed in future tools as repeated patient assays are used to assess temporal outlier effects such as those that occur across developmental time points in iPSC models (85) or in response to the environment.

## RNA SEQUENCING FOR EVALUATING RARE-DISEASE THERAPIES

We offer some perspectives on how RNA-seq can provide opportunities for evaluating rare-disease therapies. Currently, epidemiology and genetic data from health records and large-scale population biobanks are routinely used to identify the prevalence of a rare disease, which informs not only a potential market for a therapeutics company but also what types of approaches might be successfully employed. In many cases, accurate reporting of rare-disease prevalence can be a challenge from epidemiological and/or genetic data alone, given the different national reporting mechanisms, subclinical presentations, unidentified disease alleles, variable penetrance, and epistatic effects (120). As population-scale RNA-seq data continue to grow, direct observations of expression or splicing levels can potentially enhance our understanding of the prevalence of particular diseases, particularly as we further map the role dosage plays in rare-disease etiology. In this regard, efforts have been made to accurately estimate the sizes of genetic effects on gene expression as a means to subsequently identify physiologically relevant dosage effects (80). Further, recent efforts by Dong et al. (29) to map tissue-specific levels of dosage sensitivity using population-scale DNA sequencing and RNA-seq have identified constraints in genes based on their functional categories to subsequently better infer pathogenetic modes of inheritance and levels of gene expression.

While information about dosage informs disease etiology and prevalence, RNA-seq can also inform the targeting and efficacy of therapeutic strategies for rare diseases. Emerging RNA medicines can modulate dosage through small interfering RNAs or ASOs. For ASOs, steric blocking of splice modulators can selectively include or exclude exons to remove out-of-frame effects or induce specific isoform switching—here, RNA-seq data inform potential splice junctions to target. One example is ongoing clinical trials and FDA approval of skipping ASOs in the *DMD* gene for Duchenne’s muscular dystrophy to remove out-of-frame mutations in patients (93). Reference patient data can also confirm expected pathogenic splicing patterns in individuals with rare diseases and have helped to enable patient-customized ASO therapy in rare orphan diseases (55). But ASO strategies can also leverage reference, population-scale RNA-seq to identify cryptic or nonproductive splice products whose blocking may lead to isoform switching and increases in gene expression (68). In a study by Lim et al. (68), analysis of 78 RNA-seq samples from diverse tissues identified ~1,200 nonproductive splice events in disease-associated genes. Analysis of public data from hundreds of thousands of RNA-seq samples has since only increased the discovery of common, unannotated splicing events and potential targets (25, 97). The development of bioinformatic tools for mining both patient and population RNA-seq data for promising targets remains an ongoing challenge. Further, even when targets are identified, subsequent follow-up RNA-seq in treated cell lines has demonstrated its efficacy in identifying unwanted off-target effects (78).

Small-molecule therapies have also increasingly benefited from large-scale RNA-seq. The LINCS program has compiled more than 1.5 million gene expression signatures for more than 40,000 small-molecular perturbations (54), enabling rapid screening for gene expression signatures that reverse or mimic a disease state (33, 87, 95). However, challenges with these approaches are that they often have limitations on the range of cell types explored and that repositioning activities have focused on reversing signatures in the disease state where multiple homeostatic and protective factors could also be active. Studies that predict gene expression from genetic data provide another opportunity to identify gene expression signatures that preempt the disease state (37, 115). Typically, the prediction of gene expression is used to perform transcriptome-wide association studies (TWASs) to identify genes whose expression is correlated to disease state, in lieu of collecting gene expression data in these individuals. Song et al. (100) recently used this approach to assess computational drug repositioning for atopic dermatitis by evaluating enrichment of TWAS genes within drug signatures. However, as TWASs can generate risk and protective predicted risk profiles, they can also generate signatures that inform directions of risk. Recent approaches like PharmGWAS (53) provide multiple approaches for drug repositioning using TWAS data predicted from population-scale RNA-seq.

## FUTURE OPPORTUNITIES

A major challenge with RNA-seq compared with DNA sequencing in the context of rare-disease diagnosis is the unknown developmental and tissue specificity of disease effects. As yet, there are no convenient approaches to predict what developmental stages and cell types would be informative for a patient with an undiagnosed condition. Unlike complex diseases with multigene involvement, where heritability approaches can be a means to identify cell types and developmental time points, in rare diseases this often requires understanding the cell-type biology of a single gene. Further, detecting a disease-associated transcriptome signature may not always provide immediate insights into effective therapies as RNA signatures can have high interindividual variability and inaccuracy with respect to transcript isoform annotation, and may even be buffered at the protein-coding level (5). However, the increasing availability of long-read transcriptomic resources is expected to improve the resolution of transcript isoforms, which may in part increase the resolution for modeling the impact of variants from mRNA abundance through protein expression (39). Further, the growing availability of reference multiomics provides new signatures beyond transcriptome features alone, and recent studies have successfully utilized both methylation and proteomics to provide diagnoses for individuals with rare diseases (72). In addition, the increasing availability of reference resources across the human life span will be useful in identifying molecular profiles of disease matched for age of onset. Efforts led by the Developmental Genotype–Tissue Expression (dGTEx) consortium aim to provide large-scale reference profiles of multiple tissues across early to late childhood. Complementary to this resource, transcriptomic characterization of embryonic and fetal tissues provides opportunities to understand transcriptomic profiles of disease at the point of first emergence (14).

There are several opportunities for future computational analysis of RNA-seq data for rare-disease applications. Tools for discovery of meaningful aberrant transcriptome signatures have emerged, such as DROP and LeafCutterMD (51, 122). However, there is a need for strategies that consider the network effects of outliers, integrate data across omics layers, and integrate genetic information and phenotype information. In the context of rare-disease therapy, there are numerous new opportunities to consider how integrating rare-disease and population-scale transcriptomics can help refine understanding of disease prevalence and nominate new therapeutic options.

Since the emergence of RNA-seq, there has been rapid progress in its use for rare diseases (116), and since 2017, this progress has only accelerated in diagnostic applications. Routine use of RNA-seq in clinics will soon be possible; however, this will first require standardized workflows that indicate when RNA-seq will be useful for specific conditions or in the presence of specific prior data. Efforts to nominate RNA-seq for specific biosamples informed by patient-specific, noncoding variants of unknown significance would be one practical means of promoting RNA-seq in a clinical setting. In addition, harmonization of control RNA-seq data to establish more confident measures of outlier status will also enable clinical geneticists and genetic counselors to know how truly aberrant an observed effect is (i.e., 1 in 100 or 1 in 10,000). Finally, as RNA medicines rapidly provide patient-specific therapeutic options, the validation of molecular effects and minimization of off targets will require RNA-seq as part of the design workflow. The progress and promise of RNA-seq for rare diseases have clearly begun.
